# The complete chloroplast genome sequence of *Michelia balansae* var. *balansae* (Aug. Candolle) Dandy, a timber and spices species in Magnoliaceae

**DOI:** 10.1080/23802359.2021.1872427

**Published:** 2021-02-09

**Authors:** Yong-Kang Sima, Tao Wu, Shao-Yu Chen, Hui-Fen Ma, Jia-Bo Hao, Yu-Pin Fu, Yun-Feng Zhu

**Affiliations:** Yunnan Academy of Forestry & Grassland Science, Kunming, Yunnan, China

**Keywords:** Magnoliaceae, *Michelia balansae* var. *balansae* (Aug. Candolle) Dandy, complete chloroplast genome, phylogenetic analysis

## Abstract

*Michelia balansae* var. *balansae* (Aug. Candolle) Dandy is a timber and spices species in Magnoliaceae, native to China and Vietnam. In this paper, the complete chloroplast genome (cpDNA) and basic annotated information were reported and its phylogenetic relationship with other species in Magnoliaceae was analyzed. The size of chloroplast genome of *M. balansae* var. *balansae* is 160,134 bp, which exhibited a typical quadripartite structure comprising a large single-copy (LSC) region of 88,161 bp and a small single-copy (SSC) region of 18,845 bp separated by a pair identical inverted repeat regions (IRs) of 26,564 bp each. The chloroplast genome contains 131 genes, including 86 protein-coding genes (PCGs), 37 transfer RNA (tRNA) genes and 8 ribosomal RNA (rRNA) genes. The phylogenetic analysis indicated that *M. balansae* var. *balansae* is most affinal to *M. montana* and they form a nomophyletic group with other 14 *Michelia* species. This *Michelia* clade is sister to the *Aromadendron* clade with high support. All genera mentioned in this analysis are nomophyletic under the system of Magnoliaceae by Sima and Lu.

*Michelia balansae* (Aug. Candolle) Dandy is the type species of *Michelia* sect. *Dichlamys* Dandy in the family Magnoliaceae (Dandy 1974). It has two varieties, var. *balansae* (Aug. Candolle) Dandy and var. *appressipubescens* Y. W. Law. The latter variety occurs in Guizhou, Hainan and Yunnan of China (Law 1985; Law and Wu 1996; Sima and Lu 2009). *Michelia balansae* var. *balansae* (Aug. Candolle) Dandy is native to China (Fujian, Guangdong, Guangxi, Hainan, Yunnan) and Vietnam (Bac Ha, Cao Bang, Ha Giang, Ha Tay, Hanoi, Hoa Binh, Lao Cai, Nghe An, Phu Tho, Quang Binh, Son La, Thai Nguyen, Thanh Hoa, Tuyen Quang, Vinh Phuc, Yen Bai) (Law 1985; Law and Wu 1996; Sima and Lu 2009; Tran 2012; Vu and Xia 2012), and cultivated as an ornamental tree species in Southern China (Ma and Jiao 2008; Wang et al. 2013). It is a good tree species for timber and spices in tropical region and used for medicinal purpose (Nguyen et al. 2005; Tran 2012; Vu and Xia 2012). However, there has been no report on chloroplast genome information of *Michelia balansae* var. *balansae* (Aug. Candolle) Dandy until now.

The complete sequence of chloroplast genome of *Michelia balansae* var. *balansae* (Aug. Candolle) Dandy was reported in this study. The fresh leaf of *Michelia balansae* var. *balansae* (Aug. Candolle) Dandy was collected from Kunming Arboretum, Yunnan Academy of Forestry & Grassland Science (formerly Yunnan Academy of Forestry), Yunnan Province of China (25°9′4″N, 102°44′45″E). The sheets of vouchered specimen, Y. K. Sima & S. Y. Chen 99275, are stored at the herbaria, YAF and YCP. Total genomic DNA was extracted from fresh leaves using DNA Plantzol Reagent (Invitrogen, Carlsbad, CA, USA) to construct chloroplast DNA libraries. The extracted DNA was sequenced by Illumina HiSeq Sequencing System (Illumina, San Diego, CA) and shotgun library was constructed. About 1.7 Gb pair-end (150 bp) raw reads were obtained and the low-quality sequences were filtered using CLC Genomics Workbench v8.0 (CLC Bio, Aarhus, Denmark) to get high-quality clean reads. NOVOPlasty software (Dierckxsens et al. 2017) was used to align and assemble cp genome with *Pachylarnax sinica* (Y. W. Law) N. H. Xia & C. Y. Wu (JX280400) served as the reference. The complete chloroplast genome of *Michelia balansae* var. *balansae* (Aug. Candolle) Dandy was automatically annotated using CpGAVAS2 (Shi et al. 2019) and then adjusted and confirmed with Geneious 9.1 (Kearse et al. 2012). The sequence data were deposited into GenBank. Then, the complete chloroplast genome was submitted to the GenBank under the accession number of MT654130.

The size of chloroplast genome of *Michelia balansae* var. *balansae* (Aug. Candolle) Dandy is 160,134 bp, which exhibited a typical quadripartite structure comprising a large single-copy (LSC) region of 88,161 bp and a small single-copy (SSC) region of 18,845 bp separated by a pair of identical inverted repeat regions (IRs) of 26,564 bp each. The chloroplast genome contains 131 genes, including 86 protein-coding genes (PCGs), 37 transfer RNA (tRNA) genes and 8 ribosomal RNA (rRNA) genes.

In order to determine the phylogenetic position of *M. balansae* var. *balansae* (Aug. Candolle) Dandy, 24 complete chloroplast genome sequences of the family Magnoliaceae from NCBI were aligned using MAFFT v. 7 (Sima and Lu 2012; Katoh and Standley 2013; Sima, Yu, et al. 2020). Based on the system of Magnoliaceae by Sima and Lu (2012), four species of the tribe magnolieae, *Kmeria septentrionalis* (HM775382), *Pachylarnax sinica* (JX280400), *P. yunnanensis* (KF753638) and *P. omeiensis* (MK728935) were served as the outgroup. A phylogenetic tree was built using MrBayes v3.2.7 (Ronquist et al. 2012) with a single priori GTR + GAMMA model and bootstrap values were calculated from 1000 replicates.

The results of phylogenetic analysis indicated that *Michelia balansae* var. *balansae* (Aug. Candolle) Dandy is most affinal to *Michelia montana* Blume (MN990614) and they form a nomophyletic group with other 14 species of the genus *Michelia* Linnaeus (Figure 1). This clade of the genus *Michelia* Linnaeus is sister to the clade of the genus *Aromadendron* Blume with high support and it is the same as those results of other studies (Chen, Wu, Fu, et al. 2020; Chen, Wu, Ma, et al. 2020; Sima, Li, et al. 2020; Sima, Wu, et al. 2020). All genera mentioned in this analysis are nomophyletic under the system of Magnoliaceae by Sima and Lu (2012) and it is consistent with the findings of other analyses (Chen, Wu, Fu, et al. 2020; Chen, Wu, Ma, et al. 2020; Luo et al. 2020; Sima, Li, et al. 2020; Sima, Wu, et al. 2020). The determination of the complete chloroplast genome sequence provided new molecular data to illuminate the genus *Michelia* Linnaeus in Magnoliaceae evolution.

**Figure 1.**
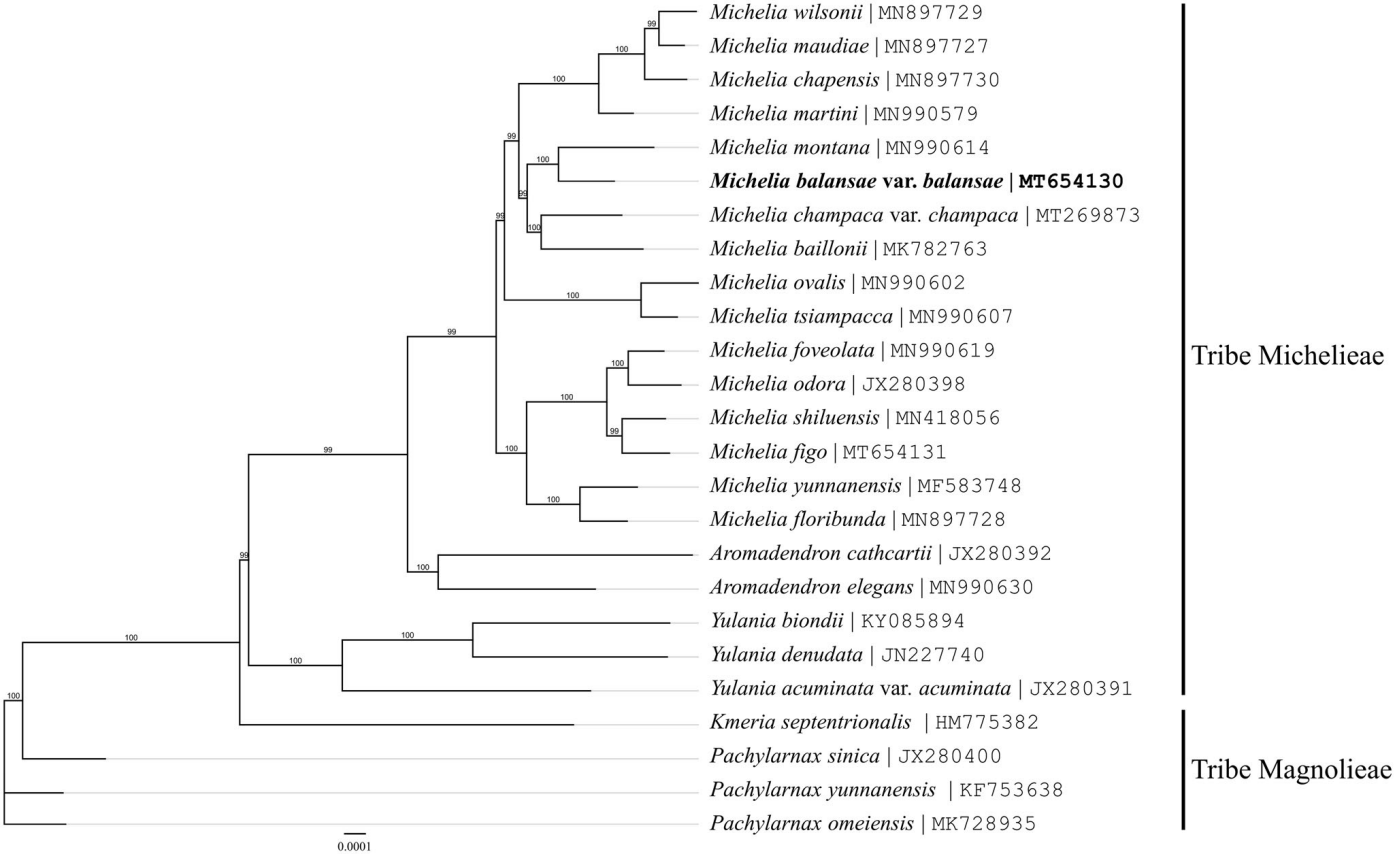
The phylogenetic tree inferred from the complete chloroplast genome sequences in the family Magnoliaceae. Bootstrap values (1000 replicates) are shown at the nodes.

## Data Availability

The genome sequence data that support the findings of this study are openly available in GenBank of NCBI at (https://www.ncbi.nlm.nih.gov) under the accession no. MT654130. The associated BioProject, SRA, and Bio-Sample numbers are PRJN682272, SRR13201444, and SAMN16984752 respectively.
